# 

**Published:** 1911-07

**Authors:** 


### INDEX.

Abstracts and Selections....12, 64, 103, 140, 186, 234, 264, 318, 352, 396, 439.................................... 483
Adenoids in School Children and Their Effect on the General System. By G. B. Taylor, M. D., Cameron, Texas....... 251
Books and Magazines......27, 151, 193, 236, 322, 357, 402, 491
Brotherhood. By G. Henri Bogart, Terre Haute, Ind...... 54
Castration of Sexual Perverts. By F. E. Daniel, M. D., Austin, Texas.................................... 369
Cold Water Cure of Inebriety. By T. D. Crothers, M. D., Hartford, Conn.................................... 43
Congenital Morphinism, With Report of Cases. By Geo. E. Petty, M. D., Memphis, Tenn.......................... 337
Correspondence ....................................63, 437
Death of Doctor Herff.............................. 473
Diseased Children. By Dr. J. N. Hurty, Secretary Indiana State Board of Health.............................. 175
Dosage. By George L. Servoss, M. D., Fallon, Nevada..... 211
Dr. Alexander S. Garrett.............................. 258
Editorial Department......11, 61, 101, 131, 181, 219, 259, 307, 345, 390, 425................................ 473
Editorial Notes and Miscellany......61, 137, 185, 230, 316, 350, 391, 435................................. 479
Editorialets ........................................11, 263
Eye Strain on the Nervous System. By G. B. Taylor, M. D., Cameron, Texas.............................. 205
Fence or the Ambulance, The.......................... 471
Hemorrhoids—Their Treatment by the Injection Method. By C. H. Wilkinson, M. D., Galveston, Texas........... 245
Holiday Reminiscences of Hawaii. By John C. Warbrick, M. D., Chicago.................................... 83
How Can We Hold Our Husbands? or Suggestions for the Correction of the "Divorce Evil." By a Wife and Mother..... 457
Hygienic Legislation. By G. Henri Bogart, M. D., Terre Haute, Ind........................................ 89
Letter to the Editor.................................. 127
Man as a Chemical Unit. By John C. Warbrick, M. D., Chicago, Ill........................................ 6
Medicine in 1950—A Look Forward. By W. T. Marrs, Peoria, Ill......................................... 336
One Bane of Prudery. By Dr. G. Henri Bogart, Terre Haute, Indiana ....................................... 165

Pneumonitis .......................................... 176
Politics in the State Medical Association.................. 476
President's Address, McLennan County Medical Society. By M. B. Saunders, M. D.................................. 50
Publisher's Department....30, 73, 118, 160, 193, 237, 280, 326, 362, 407, 450................................. 492
Pulmonary Consumption Cured by Persistent Walking, and Without Medicine. (By a Texas Physician Long Retired from Practice)....................................... 413
Puerperal Eclampsia. By L. H. Reeves, M. D., Decatur, Texas ................................................ 55
Report of a Case of Early Bone Lesion in Leprosy, with Remarks and Radiograms. By R. H. L. Bibb, M. D., Saltillo, Mexico ............................................. 1
Soft Drinks Containing Poisons. By Lyman F. Kebler, M. D., Ph. D., Chief Division of Drugs, U. S. Department of Agriculture ........................................... 8
Some Ideas on the Treatment and Management of Typhoid Fever, and Other Diseases, in Ante-Bellum Days. By a Retired Texas Physician.............................. 465
Some Obervations on the Treatment of Delayed Union of Bones. By L. Sexton, B. S., M. D., Tulane University... 123
Some Practical Points in the Prevention of Pulmonary Tuberculosis. By William Lee Secor, Ph. D., M. D., Kerrville, Texas ................................................ 86
Sterilization of the Unfit. By Dr. G. Henri Bogart, Paris, Ill...................................................... 385
Sterilizing the Unfit. By Dr. G. Henri Bogart, Paris, Ill...327
September's Farewell ................................. 178
The Psychology of the "Red Back"........................ 336
The Rational Care and Treatment of the Insane. By A. W. Fly, M. D., Galveston................................ 295
Thoughts ............................................. 180
Tonics. By William F. Waugh, A. M., M. D., Chicago.... 96
Treatment by Vaginal Douches. By L. Sexton, M. D., Tulane ................................................ 47
What Is the Cause of Appendicitis and Is the Disease on the Increase? By Henry K. Leake, A. M., M. D., Dallas, Texas ................................................ 287
Woman. By A. W. Fly, M. D., State Board of Health, Galveston, Texas.......................................... 209
Ye Editor's Outing.................................... 130

### Sentiment.

---

Embraced under the term "sentiment" are all the virtue of both head and heart. It is beautiful and ennobling. It makes one's life sublime. It prompts to all good and noble thoughts, deeds and acts. It is sentiment that controls the conduct of all good men and gives them their real character. It alone brings comfort in life, solace in adversity and hope at the hour of death. It embraces the virtues of pity, mercy, love, affection, kindness, charity, friendship, hope, faith, reverence, religion,—and that sympathy a touch of which makes all the world akin. It is belief, transcendentalism; faith, as opposed to empirical science (knowledge), and is represented by man's emotional nature. Sentiment gives us all the poetry and romance of life. It scatters flowers along the pathway and makes life's road less rugged. It is to man what the perfume is to the flower. It furnishes the theme of the poet, the painter, the sculptor, and is the basis of our polite literature—song and story. A man destitute of these principles is a monster. In our schools and by every hearthstone the cardinal virtues of pity, mercy and charity are, or should be, inculcated. They make a man's real character and stamp the seal of a community's morals. Cruelty to the humbler animals, the brutes and the helpless and unfortunate of our kind are evils that are not, and should not be, tolerated.

FERDINAND EUGENE DANIEL.

Austin, Texas.

